# Reconstitution of the Anti-Apoptotic Bcl-2 Protein into Lipid Membranes and Biophysical Evidence for Its Detergent-Driven Association with the Pro-Apoptotic Bax Protein

**DOI:** 10.1371/journal.pone.0061452

**Published:** 2013-04-23

**Authors:** Marcus Wallgren, Martin Lidman, Anders Pedersen, Kristoffer Brännström, B. Göran Karlsson, Gerhard Gröbner

**Affiliations:** 1 Department of Chemistry, Umeå University, Umeå, Sweden; 2 Swedish NMR Centre, University of Gothenburg, Gothenburg, Sweden; 3 Department of Medical Biochemistry and Biophysics, Umeå University, Umeå, Sweden; Institute of Enzymology of the Hungarian Academy of Science, Hungary

## Abstract

The anti-apoptotic B-cell CLL/lymphoma-2 (Bcl-2) protein and its counterpart, the pro-apoptotic Bcl-2-associated X protein (Bax), are key players in the regulation of the mitochondrial pathway of apoptosis. However, how they interact at the mitochondrial outer membrane (MOM) and there determine whether the cell will live or be sentenced to death remains unknown. Competing models have been presented that describe how Bcl-2 inhibits the cell-killing activity of Bax, which is common in treatment-resistant tumors where Bcl-2 is overexpressed. Some studies suggest that Bcl-2 binds directly to and sequesters Bax, while others suggest an indirect process whereby Bcl-2 blocks BH3-only proteins and prevents them from activating Bax. Here we present the results of a biophysical study in which we investigated the putative interaction of solubilized full-length human Bcl-2 with Bax and the scope for incorporating the former into a native-like lipid environment. Far-UV circular dichroism (CD) spectroscopy was used to detect direct Bcl-2-Bax-interactions in the presence of polyoxyethylene-(23)-lauryl-ether (Brij-35) detergent at a level below its critical micelle concentration (CMC). Additional surface plasmon resonance (SPR) measurements confirmed this observation and revealed a high affinity between the Bax and Bcl-2 proteins. Upon formation of this protein-protein complex, Bax also prevented the binding of antimycin A_2_ (a known inhibitory ligand of Bcl-2) to the Bcl-2 protein, as fluorescence spectroscopy experiments showed. In addition, Bcl-2 was able to form mixed micelles with Triton X-100 solubilized neutral phospholipids in the presence of high concentrations of Brij-35 (above its CMC). Following detergent removal, the integral membrane protein was found to have been fully reconstituted into a native-like membrane environment, as confirmed by ultracentrifugation and subsequent SDS-PAGE experiments.

## Introduction

Apoptosis is one of the main types of programmed cell death, and plays a crucial role in multicellular organisms by preserving tissue homeostasis and removing infected and harmful cells [1], [2]. Cells undergoing apoptosis display a number of distinctive morphological characteristics, including condensation of the chromatin and fragmentation into apoptotic bodies prior to phagocytosis [2]. There are two major apoptotic pathways: the extrinsic pathway, which is mediated by the death receptors following their activation by extracellular signals (e.g. hormones); and the intrinsic pathway, which is triggered by intracellular stress factors such as DNA damage, growth factor deprivation and reactive oxygen species [3], [4]. The intrinsic pathway, also known as the mitochondrial pathway, is mediated by proteins of the B-cell CLL/lymphoma-2 (Bcl-2) family, which tightly regulate the integrity of the mitochondrial outer membrane (MOM) [5]. When activated, the pro-apoptotic Bcl-2-associated X protein (Bax) causes mitochondrial outer membrane permeabilization (MOMP), resulting in the release of apoptogenic factors (e.g. cytochrome c) from the intermembrane space. Non-activated Bax is usually found in the cytosol in healthy cells but translocates to the MOM when activated by apoptotic stimuli [6]. At the MOM, activated Bax forms homo-oligomers that function as pores, resulting in MOMP. Its effects are counteracted by those of Bcl-2, the anti-apoptotic founding member of the Bcl-2 family that is believed to block the action of Bax and thereby promote cell survival [7], [8]. This is desirable in maintaining normal cellular homeostasis but is detrimental to the treatment of tumor cells. Bcl-2 is an integral membrane protein that resides in the MOM [9] and has been implicated as a key factor in promoting tumor cell survival and drug resistance in many forms of cancer [10]–[12].

It is not currently known how Bax and Bcl-2 interact to regulate the integrity of the MOM. One suggestion is that Bcl-2 blocks apoptosis by directly binding to and sequestering Bax [13]. However, other results imply that Bcl-2 prevents cell death by inhibiting BH3-only proteins that would otherwise activate the pro-apoptotic protein [14]. Photocrosslinking and co-immunoprecipitation assays have provided evidence that is consistent with binding between Bax and Bcl-2 [15]–[17], but there are currently no biophysical data to confirm these observations for the full-length proteins. All biophysical studies on the function and regulatory activities of Bcl-2 that have been conducted to date have examined truncated and/or otherwise mutated Bcl-2 variants [18]–[21]. In this paper, we present the results of biophysical studies on the interaction of full-length, detergent-solubilized Bcl-2 with its counterpart Bax, and also on its incorporation into native-like membrane environment upon detergent removal.

Far-UV circular dichroism (CD) spectroscopy was used to investigate protein-protein interactions in the presence of low concentrations of polyoxyethylene-(23)-lauryl-ether (Brij-35), below its critical micelle concentration (CMC). The putative interplay between Bax and Bcl-2 was further investigated by fluorescence spectroscopy using an inhibitory ligand (antimycin A_2_) that binds to Bcl-2. Bax was able to competitively prevent the binding of the ligand to Bcl-2, suggesting that the association between the two proteins is stronger than the protein-ligand interaction. Furthermore, this protein-protein association was clearly buttressed by surface plasmon resonance (SPR) measurements, in which Bcl-2 exhibited a high binding affinity to Bax in a concentration dependent manner. In addition to the protein-protein interaction study, a method for reconstituting Bcl-2 into 1,2-dimyristoyl-*sn*-glycero-3-phosphocholine (DMPC)-liposomes was developed. Because Bcl-2 is membrane-bound, it has to be studied in a membrane-like environment to fully understand its properties and functions. By using a mixture of the detergents Brij-35 and Triton X-100, it was possible to reconstitute Bcl-2 into proteoliposomes, which was confirmed by ultracentrifugation followed by SDS-PAGE. Overall, the results presented in this work provide compelling biophysical evidence for the putative direct interactions between Bax and Bcl-2. Furthermore, a method for reconstituting Bcl-2 into membranes is presented, which can be used to obtain important information on the protein's properties in its native environment. This information will be crucial both for understanding the regulation of the intrinsic apoptotic pathway and for developing novel anticancer drugs.

## Materials and Methods

### Materials

Antimycin A_2_, Triton X-100, and all chemicals and enzymes needed for cell-free protein synthesis were obtained from Sigma-Aldrich (St. Louis, MO, USA) with the exception of creatine kinase, creatine phosphate and *E. coli* tRNA extract, which were acquired from Roche (Basel, Switzerland). T7 RNA polymerase was prepared from the original pAR1219 clone. Brij-35 was purchased from Anatrace (Maumee, OH, USA). His_6_-tagged tobacco etch virus (TEV) protease was obtained as described by S. van den Berg et al. [22]. DMPC and Bio-Beads were purchased from Avanti Polar Lipids (Alabaster, AL, USA) and Bio-Rad Laboratories (Hercules, CA, USA) respectively.

### Expression and purification of Bax and Bcl-2

Bax was expressed and purified according to a previously published protocol [23]. After expressing pTYB1-Bax expression plasmid (a kind gift from Dr. Motoshi Suzuki (NIH, USA)) in BL21(DE3), the cells were mechanically lysed using a French press. The clarified cell lysate was then loaded onto a chitin affinity column. Intein self-cleavage was induced by flushing the column with buffer containing 40 mM DTT followed by incubation for 48 h. Bax was further purified by anion exchange chromatography using a mono-Q column (GE Healthcare) and size exclusion chromatography using a HiPrep 16/60 Sephacryl S-100 HR column (GE Healthcare). Cell-free synthesis of the full-length Bcl-2 was performed as previously described [24], [25], but with the addition of 0.1% w/v Brij-35 instead of polyoxyethylene-(20)-cetyl-ether (Brij-58) during the expression. For the subsequent purification steps 0.05% w/v Brij-35 was used. His_6_-Bcl-2 was expressed in batch-mode in an Eppendorf Thermomixer at 30°C for 150 minutes with agitation at 800 rpm. Once this process was complete, the reaction mixture was spun at 22000 g for 5 min prior to collection of the supernatant. His_6_-Bcl-2 was then purified by Ni-NTA agarose chromatography and cleaved using His_6_-TEV protease. After incubation overnight, the protein solution was transferred to a second Ni-NTA agarose-column and the flow-through containing the pure Bcl-2 devoid of the His_6_-tag was collected. The purity of the isolated Bax and Bcl-2 was determined by SDS-PAGE, and the yield of each was estimated from their absorption at 280 nm. Pure protein samples containing 20% v/v glycerol were flash-frozen with liquid nitrogen and stored at −80°C.

### Far-UV CD spectroscopy

The secondary structures of Bax and Bcl-2 were studied by analyzing CD spectra obtained on a JASCO J-810 Spectropolarimeter (Japan) equipped with a Peltier element for temperature control. The concentrations used were 5 µM for both Bax and Bcl-2, in 25 mM sodium phosphate (pH 7.4), 50 mM NaCl, 1 mM EDTA, 1 mM DTT and either 0.05 or 0.0028% w/v Brij-35. Spectra were acquired using a cuvette with a path length of 0.1 cm at a bandwidth of 2 nm and a scan rate of 50 nm/min, and generated by averaging 8 scans for each sample. Buffer and detergent backgrounds were subtracted from the protein signals. Temperature unfolding measurements were performed using the same conditions as were used for structure determination. The CD signal was monitored at 222 nm and the scan rate was 1°C/min.

### Antimycin A_2_ binding assay

The binding of antimycin A_2_ to Bcl-2 and Bax was investigated by fluorescence spectroscopy, using a JASCO FP-6500 Spectrofluorometer (Japan). The samples were prepared in 25 mM sodium phosphate (pH 7.4), 50 mM NaCl, 1 mM EDTA, 1 mM DTT and either 0.05 or 0.0028% w/v Brij-35. The concentrations of antimycin A_2_ and the proteins were 2.5 µM. To ensure binding equilibrium was reached, the samples were incubated overnight at 4°C. The cuvette path length was 1 cm and the excitation wavelength was set to 335 nm. Emissions were recorded between 350 and 500 nm, at 23°C.

### SPR

Sensorgrams were recorded at 25°C using a Biacore 3000 instrument (GE Healthcare). 850 nM Bax was immobilized by amine-coupling to a CM-5 chip (GE Healthcare) at pH 5.0, according to the manufacturer's instructions. Bcl-2 was then injected at 50, 25, 10, 5 and 2.5 nM concentrations at a flow rate of 20 µl/min, and the interaction experiments were performed in a running buffer containing 25 mM sodium phosphate (pH 7.4), 150 mM NaCl, 1 mM EDTA, 1 mM DTT and 0.0028% w/v Brij-35. Sensorgrams were corrected for non-specific interactions by subtracting the binding to an ethanolamine deactivated surface. The dissociation constant for the interactions between Bax and Bcl-2 was determined by fitting the response at the end of the association phase to a single-site binding equation, Y = B_max_·X/(X+K_D_). Y is the SPR Signal at equilibrium for each injection, B_max_ the plateau value of maximum binding, X the concentration of Bcl-2 and K_D_ the dissociation constant.

### Reconstitution of Bcl-2 into DMPC-vesicles

DMPC-vesicles were prepared as a 14.75 mM lipid suspension in a buffer solution containing 25 mM sodium phosphate (pH 7.4), 50 mM NaCl, 1 mM EDTA, 1 mM DTT, followed by sonication in a bath-type sonicator. Mixed micelles were prepared by solubilizing DMPC-vesicles through the addition of Triton X-100 (10% w/v solution) until the solution became clear (0.62% w/v Triton X-100). A stock solution of Bcl-2 (89.2 µM Bcl-2 solubilized with 0.05% Brij-35) was added to the mixed micelles at a 1∶200 protein-to-lipid ratio. Finally, the mixture was diluted with buffer to a final concentration of 26.8 µM Bcl-2, 5.35 mM DMPC, 0.24% w/v Triton X-100 and 0.015% w/v Brij-35. The detergent in the mixed protein lipid micelles was removed by daily incubation with Bio-Beads SM-2 Adsorbents at 4°C. Proteoliposomes were obtained after six days and isolated by ultracentrifugation against a 30% w/v sucrose barrier using a Beckman TLA 100.2 rotor operated at 213000 g for 45 min at 4°C. The isolated interfacial fraction (25 µl) was resuspended in 2 ml of buffer and spun at 15000 g for 50 min in a bench-top centrifuge, after which the supernatant was removed. The proteoliposome pellet was then resuspended in buffer and analyzed together with the supernatant and the isolated interfacial fraction by SDS-PAGE.

## Results and Discussion

### CD and ligand-binding experiments at detergent concentration above CMC_Brij-35_

**Figure 1:**
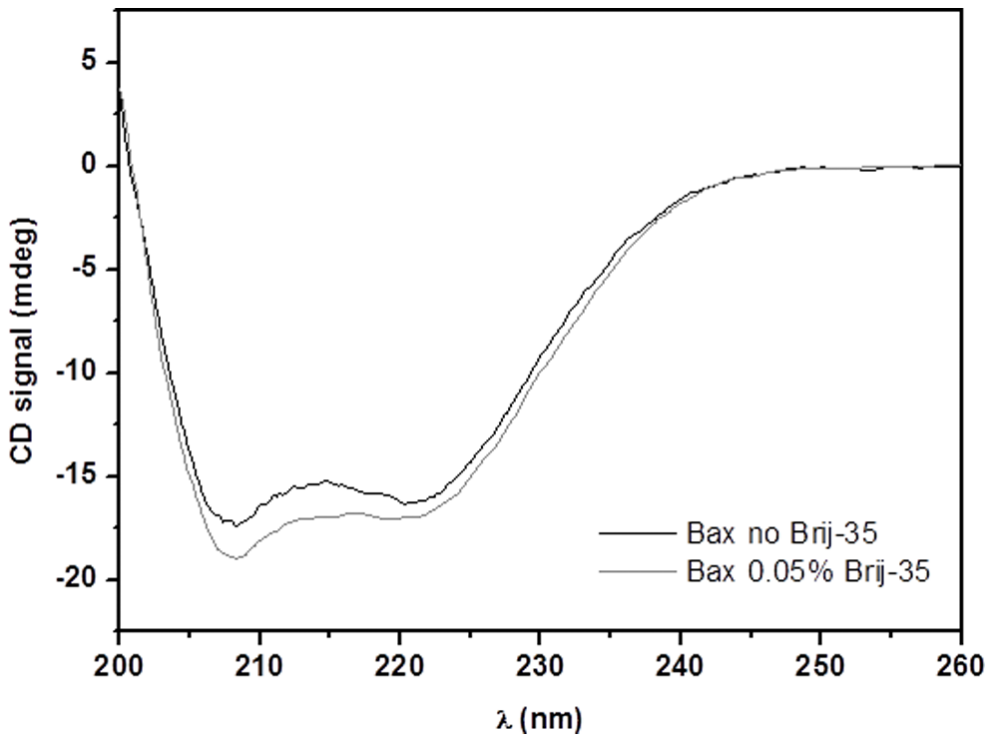
Secondary structure changes of Bax at detergent concentration above CMC_Brij-35_, probed by far-UV CD. Spectra of 5 µM Bax in the absence (–) and presence (–) of 0.05% Brij-35, in 25 mM sodium phosphate (pH 7.4), 50 mM NaCl, 1 mM EDTA, and 1 mM DTT at 20°C.

**Figure 2:**
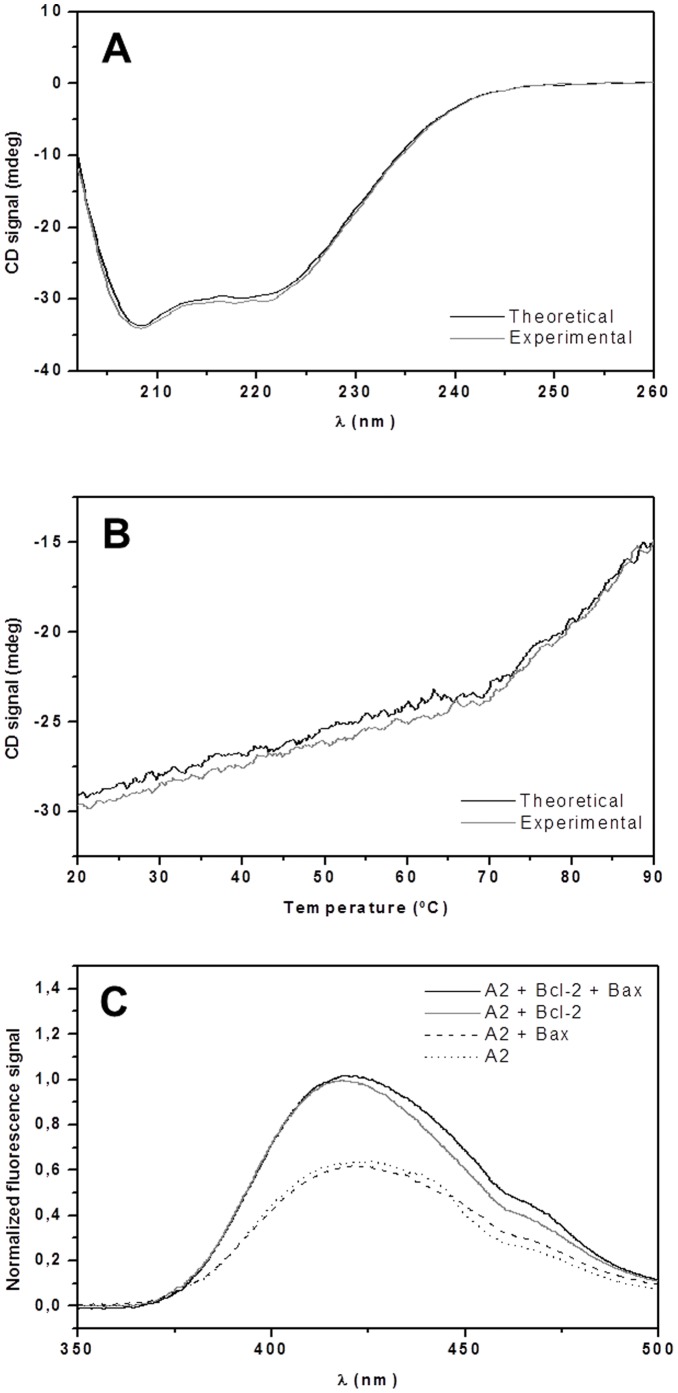
Studying the interplay between Bax and Bcl-2 at detergent concentration above CMC_Brij-35_. (**A**) Far-UV CD spectra of a solution containing 5 µM Bax+5 µM Bcl-2 (experimental, (–)) and the sum of their individual spectra (theoretical, (–)), in the presence of 25 mM sodium phosphate (pH 7.4), 50 mM NaCl, 1 mM EDTA, 1 mM DTT and 0.05% Brij-35, at 20°C. (**B**) Thermal unfolding of 5 µM Bax+5 µM Bcl-2 (experimental, (–)) compared to the sum of the melting diagrams for the individual proteins (theoretical, (–)). (**C**) Normalized fluorescence spectra of 2.5 µM antimycin A_2_ alone (··) and when incubated with 2.5 µM Bax (­­), 2.5 µM Bcl-2 (–) or both Bax and Bcl-2 (–), at 23°C. The composition of the buffer used in experiments (**B**) and (**C**) was the same as for (**A**).

Far-UV CD spectroscopy was used for the initial biophysical characterization of the interactions between the detergent-solubilized anti-apoptotic Bcl-2 protein and the pro-apoptotic Bax protein. Dlugosz et al. reported that Bcl-2 undergoes conformational changes in order to sequester and inhibit tBid-activated Bax at the mitochondrial outer membrane [26]. Bax is known to be fully activated when incubated with certain nonionic detergents (e.g. Triton X-100, Nonidet P-40) at concentrations above their CMC [27]. In addition, the pro-apoptotic protein is also able to form hetero-dimeric complexes with Bcl-2 in the presence of these detergents [17], as shown by co-immunoprecipitation [13], [16], [17] and photocrosslinking [15] assays. The nonionic Brij-35 was used to keep the Bcl-2 membrane protein in solution following cell-free expression and could potentially also activate Bax, giving rise to protein-protein interactions. Although Hsu and Youle reported that a solution of Bax in 0.2% Brij-35 (CMC_Brij-35_ = 0.011% w/v) showed no evidence of homo-dimerization, hetero-dimerization with Bcl-X_L_ or 6A7 epitope exposure [28], the intensity of the Bax CD signal increased when the level of detergent in solution was 0.05% w/v (Fig. 1). Similar increase in CD signal has previously been reported for detergent-activated Bax [29]. This discrepancy might have been due to the defined system of pure proteins used in this work, as opposed to the systems obtained by co-immunoprecipitation of cell lysates that were used in the previous study [28]. It therefore seemed reasonable to suggest that the protein might have adopted a somewhat different conformation here and potentially been activated in the presence of Brij-35. Next, to investigate whether the secondary structure (and potentially also the conformation) of the proteins changes as a consequence of any existing protein-protein interactions, the CD profiles of both proteins present were compared to the summed spectra for the individual proteins. As can be seen in Fig. 2A, the two spectra did not differ greatly, indicating that any protein-protein interaction that may have occurred did not have a significant effect on the secondary structure of either species. In addition, the sum of the melting patterns obtained when the two proteins were unfolded thermally in isolation were similar to the one observed when they were unfolded together (Fig. 2B), confirming that CD alone was not sufficient to provide unambiguous data on the putative protein-protein interaction.

To further study the interplay between Bcl-2 and Bax, a ligand-binding assay was performed. Computer-aided docking of antimycin A_3_ into the solution structure of the anti-apoptotic Bcl-X_L_ [30] indicated that it may interact with the protein's hydrophobic pocket [21]. Due to the high sequence homology between Bcl-X_L_ and Bcl-2, the crystal structure of Bcl-X_L_ [31] was used as a template for the molecular docking of antimycin A_3_ to Bcl-2 [20]. Fluorescence spectroscopy studies on truncated protein variants have previously been shown that both antimycin A_3_ and its analogue antimycin A_2_ seem to bind to the hydrophobic pocket of recombinant, truncated Bcl-2. [18], [20], [21]. Moreover, it was found that this interaction was inhibited in the presence of peptides derived from the BH3-domain of both the multidomain effector protein Bak (same function as Bax) [20] and the BH3-only protein Bad [18]. These results support a model in which Bcl-2 can sequester and inhibit Bax by binding to the BH3-domain of the pro-apoptotic protein. To investigate whether the recombinantly expressed full-length Bcl-2 and Bax behaved in the same way as was observed for the shorter protein variants, a fluorescence-based antimycin A_2_-binding assay was performed.

As shown in Figure 2C, the Bcl-2 protein clearly bound to antimycin A_2_. This is indicated by the pronounced increase in the observed fluorescence signal, which is consistent with previous reports [18], [20], [21] and implies that the expressed full-length protein was indeed correctly folded in the micellar detergent environment. It would thus contain the functionally active binding groove formed by the conserved BH1-, BH2- and BH3-domains. In contrast, Bax was not able to bind to antimycin A_2_. This was somewhat surprising given that Brij-35 could presumably induce the activation of the protein and thus expose its hydrophobic groove. The lack of interaction could be due to a difference in the binding specificities of Bax and Bcl-2. For example, the BH3-only protein Noxa binds strongly to Mcl-1 but not to Bcl-X_L_ [32], although both of these anti-apoptotic Bcl-2 family proteins are selective for Bak [33]. Remarkably, when both Bax and Bcl-2 were present, the fluorescence signal was still as strong as had been the case for Bcl-2 alone, indicating that Bax failed to prevent the binding of antimycin A_2_ to the Bcl-2 protein. Since Bax itself was not able to bind to the ligand, the high fluorescence signal was clearly due to the interaction between antimycin A_2_ and Bcl-2. This raised a question: why was Bax not able to interact more strongly with Bcl-2, even if with a lower affinity than the ligand? Possible explanations include: 1) Bax was not active in Brij-35, consistent with previously reported [28]; 2) the size of Bax, which is much larger than the BH3-domain peptide, precluded its binding in favor of the smaller antimycin A_2_; and 3) the fact that both proteins were associated with micelles prevented them from rapidly diffusing into close proximity with one-another and thus favored the binding of the smaller antimycin A_2_. Since all previously reported ligand-binding measurements were performed in the absence of detergents [18], [20], [21] and Bax has been shown to undergo hetero-dimerization with Bcl-X_L_ in the presence of octyl glucoside at a concentration below its CMC value [28], we performed a series of experiments using a lower, sub-CMC concentration of the Brij-35 detergent.

### CD, ligand-binding and SPR experiments at detergent concentration below CMC_Brij-35_

**Figure 3:**
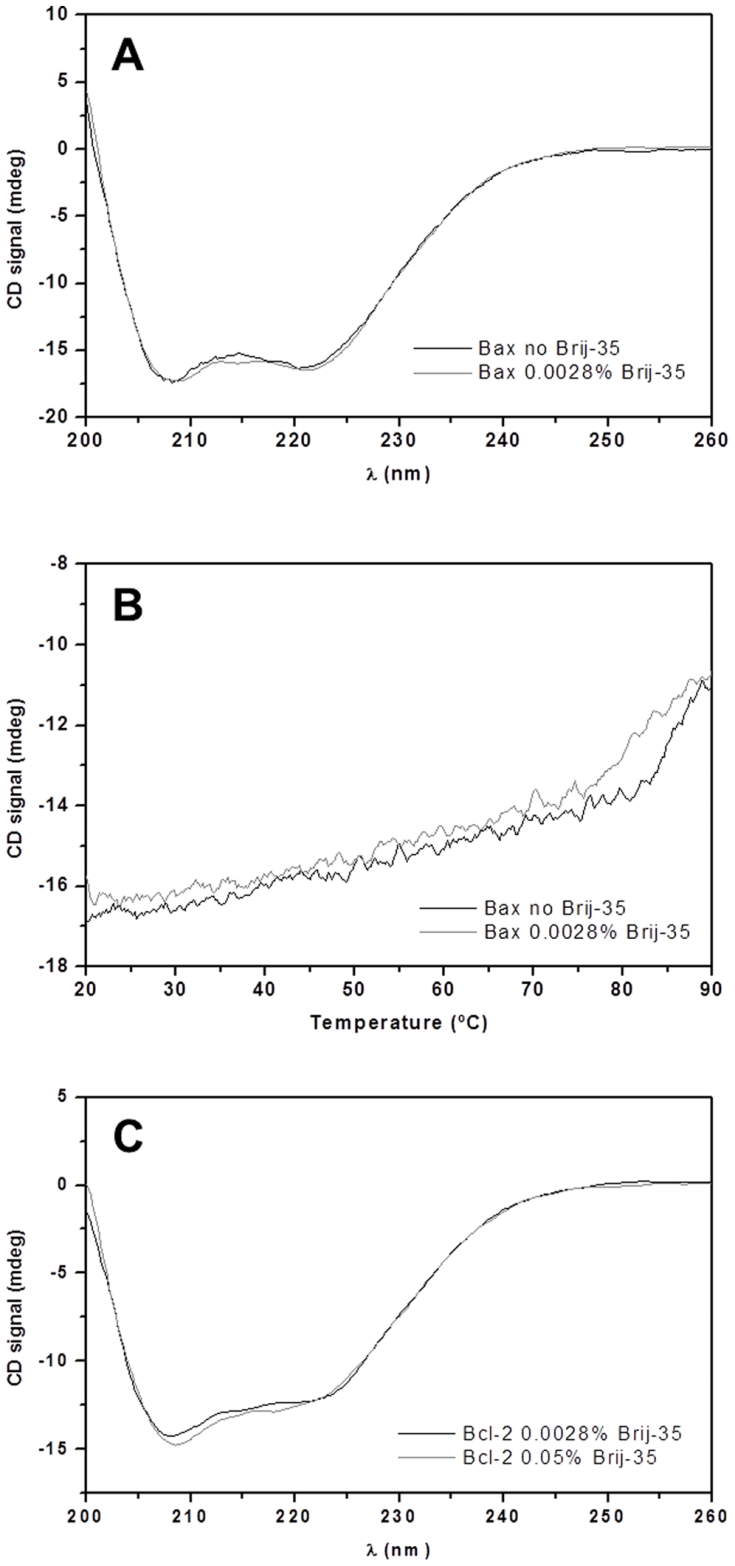
Far-UV CD spectra of Bcl-2 and Bax, and thermal unfolding of the latter, at detergent concentration below CMC_Brij-35_. (**A**) Spectra of 5 µM Bax in the absence (–) and presence (–) of 0.0028% Brij-35, in 25 mM sodium phosphate (pH 7.4), 50 mM NaCl, 1 mM EDTA, and 1 mM DTT, at 20°C. (**B**) Thermal unfolding of 5 µM Bax in the absence (–) and presence (–) of 0.0028% Brij-35. (**C**) Far-UV CD spectra of 5 µM Bcl-2 in 0.0028% (–) and 0.05% (–) Brij-35, at 20°C. The composition of the buffer used in experiments (**B**) and (**C**) was the same as for (**A**).

To investigate the effect of Brij-35 on the interaction between Bcl-2 and Bax when present at a concentration well below its CMC (0.011% w/v), CD experiments were again performed to monitor any changes in the proteins' secondary structures (Fig. 3A). The presence of the low concentration of Brij-35 did not induce any pronounced changes in the CD profile of Bax, suggesting that it had at best a modest activating effect below its CMC. However, thermal melting data for Bax indicated that the addition of Brij-35 made its thermal unfolding transition less narrow (Fig. 3B). This loss of cooperativity has previously been reported for Bax in the presence of both Bax-activating detergents and membranes [34], [35], suggesting that Bax was affected by the low concentration of Brij-35. Thus, even at a concentration below CMC_Brij-35_, Bax was presumably partially activated (i.e. not fully but to some extent changing its conformation towards the activated state) by the detergent and potentially able to interact with Bcl-2. Before further interaction studies could be performed, it was necessary to verify the structural integrity and solubility of Bcl-2 under the experimental conditions. Therefore, the secondary structure of the anti-apoptotic protein was also analyzed using CD. As can be seen in Fig. 3C, the CD signal of Bcl-2 at lower Brij-35 concentrations was slightly less intense, but the protein retained the same profile as had been observed previously, indicating that it remained correctly folded. Since there was no indication of protein aggregation, further biophysical studies were performed under these conditions.

**Figure 4:**
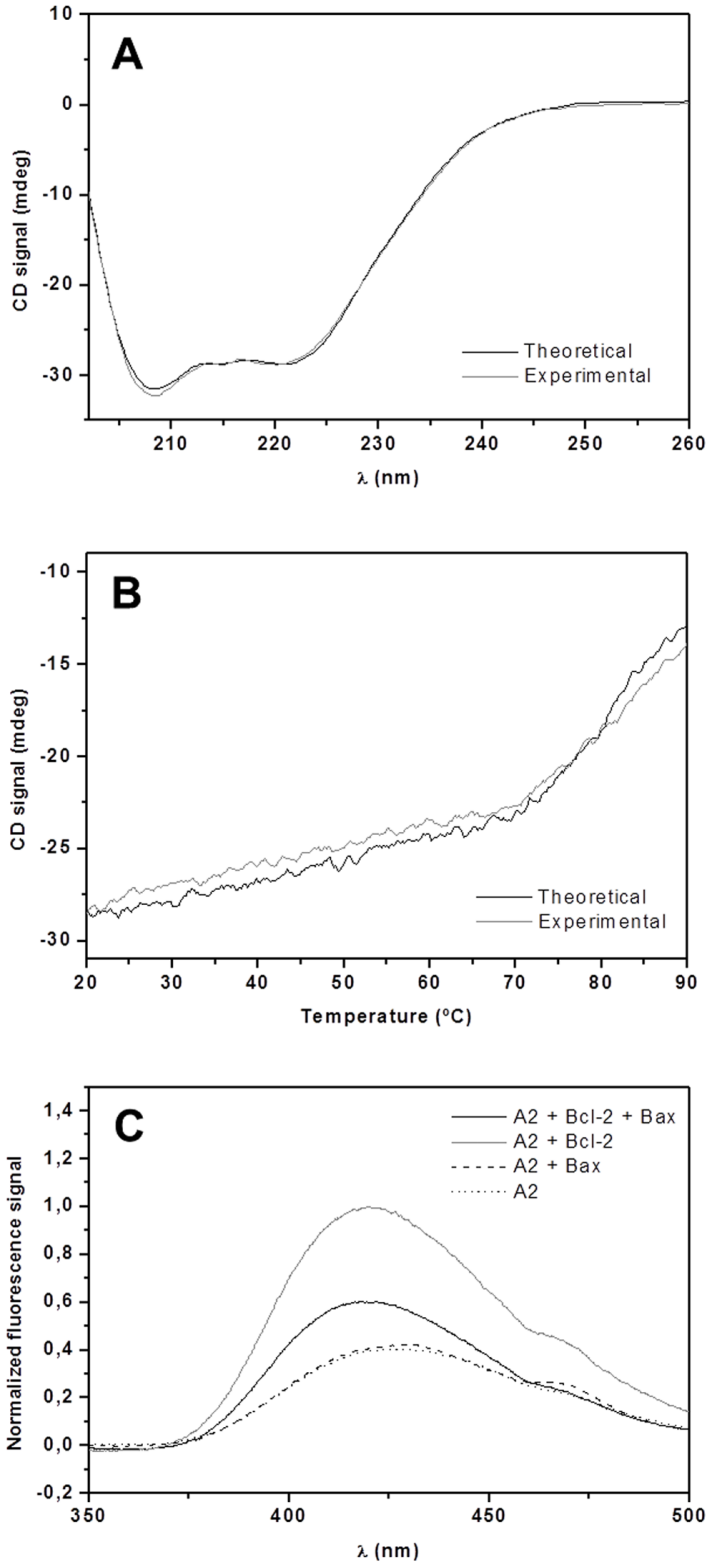
Studying the interplay between Bax and Bcl-2 at detergent concentration below CMC_Brij-35_. (**A**) Far-UV CD spectra of 5 µM Bax+5 µM Bcl-2 (experimental, (–)) and the sum of their individual spectra (theoretical, (–)), in the presence of 25 mM sodium phosphate (pH 7.4), 50 mM NaCl, 1 mM EDTA, 1 mM DTT, and 0.0028% Brij-35, at 20°C. (**B**) Thermal unfolding of 5 µM Bax+5 µM Bcl-2 (experimental, (–)), and summed diagrams for the melting of the individual proteins (theoretical, (–)). (**C**) Normalized fluorescence spectra of 2.5 µM antimycin A_2_ alone (··) and when incubated together with 2.5 µM Bax (­­), 2.5 µM Bcl-2 (–) or both Bax and Bcl-2 (–), at 23°C. The composition of the buffer used in experiments (**B**) and (**C**) was the same as for (**A**).

**Figure 5:**
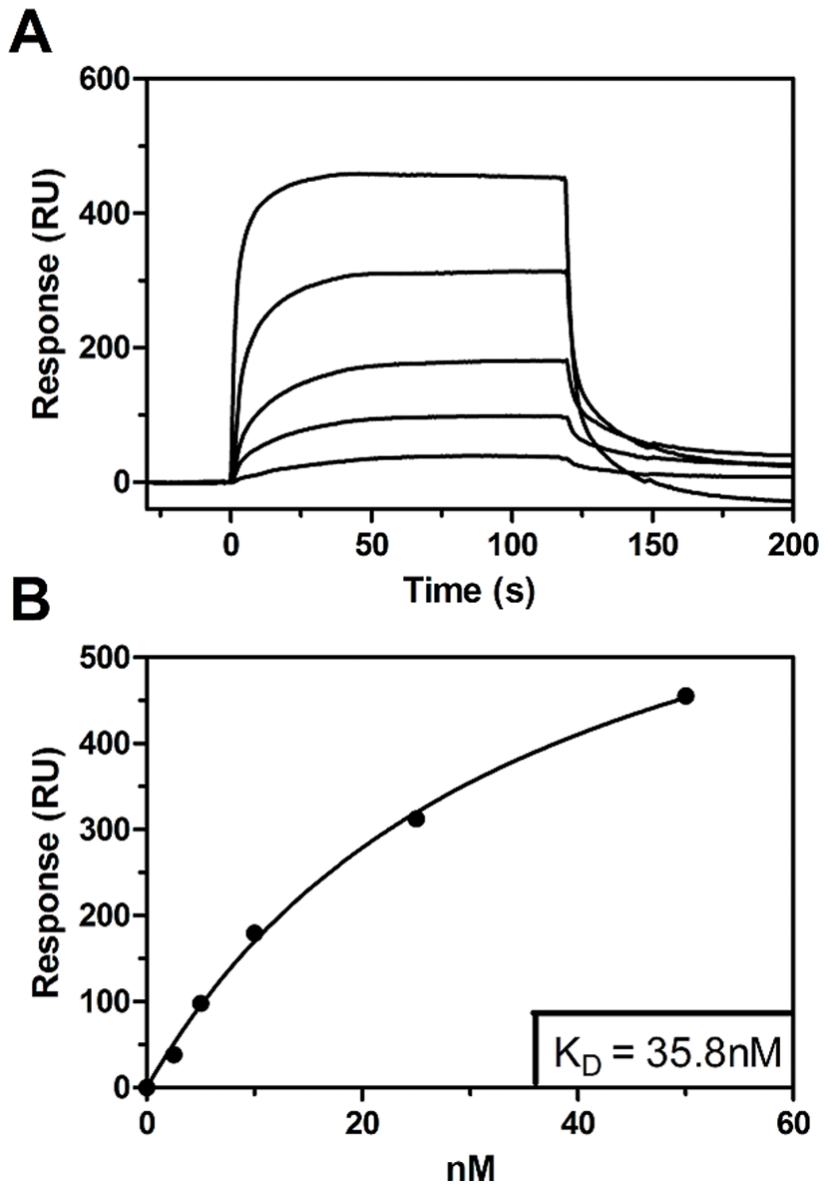
SPR sensorgrams and binding curve of the interactions between Bcl-2 and Bax at detergent concentration below CMC_Brij-35_. (**A**) Sensorgrams of Bcl-2 injected at 50, 25, 10, 5 and 2.5 nM respectively (from the top to the bottom), in 25 mM sodium phosphate (pH 7.4), 150 mM NaCl, 1 mM EDTA, 1 mM DTT and 0.0028% Brij-35 at 25°C, over a Bax-coupled surface. (**B**) Binding curve fitted to the obtained sensorgrams, yielding a K_D_ value of 35.8 nM.

The far-UV CD profile of the sample containing both proteins was very similar to that obtained by summing those for Bax and Bcl-2 alone, as had been the case for the samples incubated in 0.05% Brij-35 (Fig. 4A). However, a more pronounced discrepancy was observed in the thermal unfolding data (Fig. 4B): the sample containing both Bcl-2 and Bax exhibited less cooperative melting than would have been expected based on the summed data for the two proteins in isolation. This stands in contrast to the results obtained in the corresponding experiments using higher detergent concentrations. While this dissimilarity was not dramatic, it still provides support for the existence of a protein-protein interaction at the lower detergent concentration. As in the previous experiments, the fluorescence signal of antimycin A_2_ in the ligand-binding assays performed at lower detergent concentrations became more intense in the presence of Bcl-2. In keeping with the CD results, this suggests that Bcl-2 retains its native fold under these conditions. Strikingly, when Bax was added the intensity of the signal declined significantly, indicating that the population of ligand molecules binding to the Bcl-2 site had decreased. Since Bax alone did not influence the antimycin A_2_ signal at all, it can be concluded that Bax most likely replaced the ligand, thereby quenching the fluorescence signal of antimycin A_2_. The binding of antimycin A_2_ to its target, the Bcl-2 protein, can thus be competitively inhibited by the higher affinity of full-length Bax in the absence of interfering Brij-35 micelles, which presumably either trigger a conformational change in Bax that prevents hetero-dimerization or physically hinder its interaction with Bcl-2. Furthermore, SPR experiments performed below CMC_Brij-35_ clearly displayed a strong affinity of Bcl-2 to Bax (Fig. 5A and 5B); a concentration dependent association, which existed even at a very low concentration of added Bcl-2 (2.5 nM). Together, these results strongly support the occurrence of direct protein-protein interactions between Bcl-2 and Bax when the latter is only partially activated, which is consistent with previous studies on Bax after incubation with sub-CMC concentrations of octyl glucoside. Under these conditions, Bax did not exhibit any homo-dimerization but was still able to form hetero-dimers with Bcl-X_L_ [28]. It has previously been shown that mutations in the hydrophobic groove region of Bcl-2 aggravate its association with Bax, as indicated by co-immunoprecipitation [16]. The biophysical data presented herein therefore provide further support for the central role of the hydrophobic groove region of Bcl-2 in its interaction with Bax.

### Reconstitution of Bcl-2 into proteoliposomes

Micelles can at best partially mimic the native membrane environment of Bcl-2 and translocated Bax, but are largely insufficient to emulate the *in vivo* conditions of the mitochondrial membranes. Therefore, to fully understand how the Bcl-2 family proteins regulate apoptosis, the full-length variants have to be studied in a real membrane milieu. To accomplish this, they can be incorporated into native-like membrane systems for further biophysical analysis. Unfortunately, this process of reconstituting membrane proteins into proteoliposomes is often cumbersome and difficult to achieve. Using our previously published cell-free protein synthesis protocol, Bcl-2 was expressed and purified in the presence of Brij-58 [25]. Although this detergent was suitable for producing the protein, it could not be removed during subsequent attempts at reconstitution. It was therefore replaced with Brij-35, which belongs to the same class of detergents but has been demonstrated to be removable using Pierce columns [36]. This suggests that it could also be eliminated by other means, thus enabling the reconstitution of Bcl-2. The switch to Brij-35 did not have any significant effect on the *in vitro* production of Bcl-2 or its subsequent purification. Moreover, as demonstrated by both CD and fluorescence spectroscopy, the protein appeared to be correctly folded both at high and low detergent concentrations.

**Figure 6:**
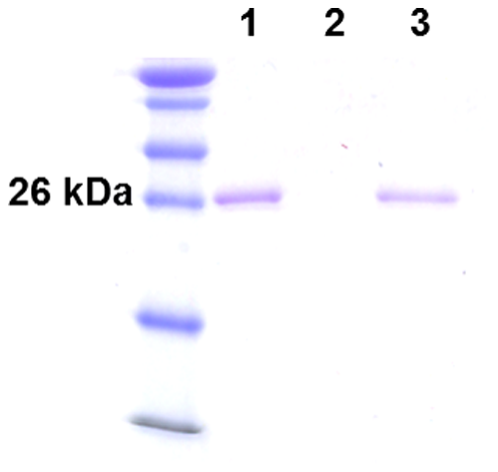
SDS-PAGE of Bcl-2 proteoliposomes. Lane 1: isolated interfacial fraction after ultracentrifugation with a 30% sucrose barrier. Lanes 2 and 3: supernatant and pellet after centrifugation of the resuspended interfacial fraction, respectively.

To successfully reconstitute a membrane protein, it is generally necessary to generate mixed detergent micelles containing lipid and protein molecules. The choice of an appropriate detergent makes it possible to incorporate the protein of interest into intact lipid bilayers in its native form when the detergent is removed, without forming a heterogeneous system or causing protein aggregation. Brij-35 alone was not able to generate mixed micelles at sufficiently low detergent concentrations (too high a concentration would impede the reconstitution process), and so Triton X-100 was also added to the lipid samples prior to incubation with the Brij-35 protein micelles to achieve satisfactory mixing. Triton X-100 can be removed using Bio-Beads [37], a property that is widely used in reconstitution studies. It was therefore considered preferable to add Triton X-100 to the sample rather than increasing the amount of Brij-35. The addition of Triton X-100 clarified the vesicle solution rapidly, indicating the formation of mixed micelles. The solution remained clear following the addition of Bcl-2 in Brij-35. After six days of daily detergent removal using batches of Bio-Beads in the cold room under gentle agitation, the protein/lipid-mixture looked the same as the detergent-free reference solution, which contained only DMPC vesicles. Since the concentration of Brij-35 in the mixture had been reduced to less than 0.05% in favor of Triton X-100, there was a risk that the solubility of Bcl-2 might have declined during the fairly long incubation period. To ensure that any proteins present in the pellet following centrifugation had been associated with the liposomes rather than merely being precipitates, the putative proteoliposomes were isolated by centrifugation using a sucrose cushion. Inspection of the centrifuged tubes containing Bcl-2 and the liposomes did not reveal any protein aggregates in the bottom layer, and most of the pellet was located close to the sucrose/water interface, as was observed for the pure DMPC reference. Following the isolation of the liposome fraction (possibly loaded with Bcl-2 molecules) from the interface, it was diluted with buffer, centrifuged again and analyzed by SDS-PAGE. The SDS gel (Fig. 6) clearly showed that Bcl-2 had associated with the liposomes during the incubation period, demonstrating its successful reconstitution into the lipid membrane environment.

## Conclusions

During the last few decades the processes involved in apoptotic cell death have been investigated intensively. Despite this, little is known about the regulation of the mitochondrial apoptotic pathway, and particularly about how the anti-apoptotic Bcl-2 family proteins inhibit their pro-apoptotic counterparts. The pro-survival Bcl-2 protein has been reported to prevent the action of the killer protein Bax, either by direct interaction or by inhibiting Bax-activating BH3-only proteins. Although several previous studies have reported direct binding between Bax and Bcl-2, no biophysical data on the interactions between the two full-length proteins has previously been presented. In this paper, we present to our knowledge the first biophysical data on the interaction between full-length Bcl-2 and Bax and provide evidence in support of their putative binding interactions. In addition, as a first step towards studying Bcl-2 in a native-like environment, the anti-apoptotic protein was reconstituted into DMPC-based proteoliposomes. This reconstitution protocol could be used in future studies to examine how full-length Bcl-2 behaves when integrated into mitochondrial membranes under conditions that simulate normal and pro-apoptotic conditions (the latter of which can be induced by the addition of oxidized phospholipids, as shown by us recently [23]). Further biophysical studies on membrane-protein and protein-protein interactions involving reconstituted Bcl-2 and Bax will provide information that will be vital for understanding the machinery of apoptotic regulation and for developing novel chemotherapeutic agents.
